# Evaluation of Exercise Tolerance in Non-obstructive Hypertrophic Cardiomyopathy With Myocardial Work and Peak Strain Dispersion by Speckle-Tracking Echocardiography

**DOI:** 10.3389/fcvm.2022.927671

**Published:** 2022-07-22

**Authors:** Ye Su, Qionghui Peng, Lixue Yin, Chunmei Li

**Affiliations:** ^1^School of Medicine, University of Electronic Science and Technology of China, Chengdu, China; ^2^Department of Cardiovascular Ultrasound, Sichuan Provincial People’s Hospital, University of Electronic Science and Technology of China, Chinese Academy of Sciences Sichuan Translational Medicine Research Hospital, Chengdu, China

**Keywords:** myocardial work, peak strain dispersion, exercise tolerance, hypertrophic cardiomyopathy, global longitudinal strain (GLS)

## Abstract

**Background:**

The aim of this study was to evaluate exercise tolerance in non-obstructive hypertrophic cardiomyopathy (HCM) by investigating the value of myocardial work (MW) combined with strain peak dispersion.

**Methods:**

A total of 65 patients with non-obstructive HCM and normal left ventricular ejection fraction were enrolled and 60 healthy subjects were selected as controls. The automated function imaging (AFI)-two-dimensional ultrasonic speckle-tracking technology was used to obtain the values for peak global longitudinal strain (GLS), longitudinal strain peak time dispersion (PSD), 18-segment systolic longitudinal peak strain (LPS), 18-segment longitudinal strain peak time (TTPLS), global waste work (GWW), global constructive work (GCW), global work index (GWI), global work efficiency (GWE), and exercise metabolic equivalents (METS).

**Results:**

(1) Values for LV-GLS (−17.77 ± 0.20 vs. −21.66 ± 0.42%) were lower and PSD (95.10 ± 8.15 vs. 28.97 ± 1.50 ms) was prolonged in patients with HCM (*p* < 0.01). (2) An increasing trend was shown in the basal segment < intermediate segment < apical segment for both patients with HCM and controls, although each segment had lower values in the HCM group. (3) TTPLS was prolonged in the HCM group (*p* < 0.01). (4) GWE, GWI, and GCW were all lower (*p* < 0.01) and GWW was higher in patients with HCM (*p* < 0.01). (5) Values of GWE were less than 92.5%, GWI less than 1,200 mmHg, GCW less than 1,399 mmHg, these abnormal values are helpful for the diagnosis of impaired exercise tolerance and poor prognosis (6) The METS and LV-GLS of HCM in the asymmetric group were significantly lower than that in AHCM group, but the PSD was significantly greater than that in the AHCM group. Values of LPS-BL (−13.13% ± 2.51% vs −10.17% ± 2.20%) in the apical HCM group were better than in the asymmetric HCM group (*p* < 0.05).

**Conclusion:**

GCW, GWI, and GWE can be safely measured by resting echocardiography to evaluate exercise tolerance in patients with HCM who cannot perform an exercise-based examination. Such measurements provide a basis for clinical decisions regarding exercise and drug prescription.

## Introduction

Many patients with non-obstructive hypertrophic cardiomyopathy (HCM) and preserved ejection fraction have no obvious symptoms at rest in the early stages, and most patients seek medical attention due to limitations on daily exercise or activities. HCM is one of the main causes of sudden death in young people following activity (1), and previous studies have estimated that its contribution to sudden cardiac death may be as high as 10.3% (2). Such statistics illustrate the urgent need for more sensitive and comprehensive techniques to detect abnormal cardiac function in patients with HCM. Subtle myocardial systolic and diastolic dysfunctions occur that are not always detectable using standard echocardiographic parameters such as left ventricular ejection fraction (LVEF). Previous studies have shown that LV global longitudinal strain (LV-GLS), measured using speckle-tracking echocardiography, is often impaired and constitutes an adverse outcome predictor in HCM patients with preserved LVEF. However, LV-GLS remains load-dependent limiting the assessment of LV function under certain hemodynamic conditions such as abnormal blood pressure. A novel speckle-tracking echocardiographic technique evaluates myocardial performance by constructing an LV pressure–strain loop (PSL) and integrating non-invasively measured arterial blood pressure and longitudinal strain. This approach allows the detection parameters of the same cardiac cycle to be measured, excludes the influence of heart rate (HR) inconsistency on the analysis, and quantitatively analyzes and evaluates myocardial function (3). The derived parameters from LVPSL can not only reflect the global and regional LV function but also reflect the synchronization and direction of LV contraction (4). Previous studies (5–7) have confirmed that non-invasively and invasively measured LVPSL have good consistency and correlation, the myocardial work (MW) assessed using non-invasive LVPSL is reduced and associated with LV fibrosis in patients with HCM. However, the relationship between MW and exercise tolerance in HCM is currently unknown. Therefore, the aims of this study were (1) to describe the global and segmental indices of MW in different HCM phenotypes and (2) to evaluate the relationship of MW with exercise tolerance.

### Research Subjects

A total of 65 patients (45 males, 20 females, mean age: 47.54 ± 1.93 years) with non-obstructive HCM, including 34 patients with apical hypertrophy and 31 patients with asymmetric hypertrophy, diagnosed in Sichuan Provincial People’s Hospital between January and December 2019, were consecutively enrolled. The inclusion criteria were as follows: presence of HCM diagnosed according to the 2017 Chinese Guidelines for the Diagnosis and Treatment of Hypertrophic Cardiomyopathy in Adults and the 2014 ESC Guidelines. The exclusion criteria were as follows: obstruction of the left or middle left ventricular outflow tract, hypertension, coronary heart disease, moderate or higher aortic stenosis, or other distinct diseases causing cardiac hypertrophy. A total of 60 age-matched healthy subjects who were followed up in Sichuan Provincial People’s Hospital in 2019 were selected as controls. Clinical data and echocardiographic parameters of all participants were collated.

### Instruments and Methods

A GE Vivid E95 color Doppler ultrasound diagnostic apparatus with 4V-D probe (frequency 1.5–4.0 MHz) (GE Medical Systems, Milwaukee, WI, United States) was used. All subjects had discontinued beta-blockers or calcium channel blockers for at least 24 h. 2 experienced sonographers Physicians performed echocardiography, by continuously acquire three-plane dynamic images of the apex for at least 4 cardiac cycles (image frame rate 60 frames/s), images were digitally stored and analyzed offline using EchoPAC (203) workstation, and performed the synchronous recording of electrocardiogram, measured resting systolic and diastolic blood pressure. All patients with HCM underwent symptom-limited treadmill exercise and electrocardiogram (ECG) using Bruce protocol immediately after resting standard transthoracic echocardiography. The study by Mendes et al. (8) included participants (112 healthy adults) with different levels of fitness, ages, and genders to increase the representation of the population and found that metabolic equivalents (METS) ≤6.8 had higher specificity and relatively higher accuracy. The authors also reported a maximal reduction in errors in activity intensity assessment, considered to be associated with reduced exercise tolerance and poor long-term prognosis. The study divided all subjects into group A (METS ≤6.8) and group B (METS >6.8) and also sorted them according to different hypertrophic segments apical HCM (AHCM), or asymmetric HCM for further statistical analysis (8).

SunTech Tango synchronized ambulatory blood pressure monitor (SunTech Medical Instruments, Morrisville, NC, United States) and Mortara X-Scribe tablet motion analysis system (Mortara Instrument, Milwaukee, WI, United States) were used to perform the symptom-limited treadmill exercise and an ECG and blood pressure during and after exercise. Before the examination, all subjects had discontinued beta-blockers or calcium channel blockers for at least 24 h. Resting systolic and diastolic blood pressure were measured, and a synchronous electrocardiogram recording was taken. All participants signed informed consent after confirming that they had no contraindications to exercise testing. The BRUCE protocol was performed at the exercise stage. When subjects reached the target HR or one of the following symptoms occurred, the exercise stopped: (1) ST-segment elevation >1.0 mm in no pathological Q-wave lead (except V1 or aVR), (2) systolic blood pressure decreased >10 mmHg with other evidence of ischemia, (3) moderate to severe angina pectoris, (4) CNS symptoms, such as ataxia, dizziness, and syncope, (5) signs of hypoperfusion, such as cyanosis and pallor, (6) persistent ventricular tachycardia, and (7) technical difficulties in checking ECG or systolic blood pressure (9). ECG, exercise blood pressure, and METS were recorded. All subjects exercised to target heart rate (THR) if no symptoms restricted exercise. THR was calculated according to the following formula: THR = 220-age.

All parameter measurements and analyses were made in accordance with the American Society of Echocardiography (ASE) guidelines (10–12). Measurements were made along the short-axis views orthogonal to the circumference of the endocardium and epicardium, wherever maximal wall thickness occurs, ensuring that the cut was not oblique to the long axes of the LV. The criteria for the diagnosis of asymmetric HCM include the following: interventricular septum or anterolateral or inferior wall thickness ≥15 mm in one or more myocardial segments. The criteria for the diagnosis of AHCM include the following: apical wall thickness ≥15 mm in one or more myocardial segments or when the ratio between apical and basal wall thickness exceeds 1.3:1.

The following parameters were measured and calculated during offline data analysis: (1) conventional parameters: left ventricular end-diastolic volume (LVEDV), left ventricular end-systolic volume (LVESV), and LVEF were measured using the Simpson method. LV dimensions such as LV septal thickness (IVS), LV posterior wall thickness (LVPW), and left atrial (LA) diameter were measured from the parasternal long-axis view, and maximum LV wall thickness was assessed from three short-axis views from base to apex to identify the different phenotypes of left ventricular hypertrophy (LVH); (2) myocardial strain: 2D strain imaging was performed by using three consecutive cardiac cycles, and speckle-tracking analysis of the LV was performed in three apical (4, 2, and 3 chambered) views. The 17 regions of interest were automatically created and manually adjusted when necessary and then obtained the parameters including GLS, longitudinal strain peak time dispersion (PSD), 17-segment systolic longitudinal peak strain (LPS), 17-segment longitudinal strain peak time (TTPLS), the basal, mid, and apical segment longitudinal systolic peak strain (LPS-BL, LPS-ML, and LPS-AL), and longitudinal strain peak time [basal segment (BLST), middle segment (MLST), and apical segment (ALST)] and then calculated by averaging the peak longitudinal strain and time of corresponding segments. (3) MW: a non-invasive LVPSL was then constructed using the software (EchoPAC 203) and adjusted according to the duration of the ejection and isovolumetric phases, which were defined by the opening and closure of the mitral and aortic valves. During the LV ejection period, the total work within the area of the LVPSL during the LV ejection period represented the myocardial global work index (GWI), the MW performed during segmental shortening represented global constructive work (GCW), and the MW performed during segmental elongation represented global waste work (GWW) and calculated as the averages of each LV segment values according to the 17-segment model. Global cardiac efficiency (GCE) was an average of all segmental values expressed as GCW/(GCW + GWW) × 100%. (4) Exercise METS.

### Statistical Methods

All statistical analyses were performed using the SPSS version 23.0 software (IBM SPSS Statistics, version 23). Continuous variables are expressed as means ± standard deviations. Comparisons between groups were made by two independent samples *t*-tests, and a value of *p* < 0.05 was considered to be statistically significant. Multivariate linear regression analysis was used to identify independent factors indicating exercise intolerance in patients with HCM. The sensitivity and specificity of variables for the prediction of exercise intolerance were identified by receiver operating characteristic (ROC) curves.

## Results

1. Among the 65 patients, 31 cases had mild mitral regurgitation, 11 had moderate mitral regurgitation, 2 had severe mitral regurgitation, 28 had mild tricuspid regurgitation, and 10 had moderate tricuspid regurgitation. All subjects with HCM included experienced non-obstructive HCM but 20 patients had increased outflow tract velocity.
2. Comparison of general information and conventional echocardiographic parameters (Table 1).

**TABLE 1:** Comparison of general information.

Variable	Normal group	HCM group	*P*
Age (year)	46.75 ± 1.76	47.54 ± 1.93	0.079
EDV (ml)	108.89 ± 4.31	77.61 ± 5.17	0*
ESV (ml)	26.02 ± 1.92	27.89 ± 1.90	0.376
EF (%)	73.33 ± 6.49	69.81 ± 5.99	0.014*
SBP (mmHg)	125.21 ± 9.79	142.05 ± 1.30	0.342
DBP (mmHg)	75.32 ± 1.30	75.85 ± 1.06	0.729
BSA (m^2^)	1.78 ± 0.02	1.86 ± 0.03	0.027*
HR (bpm)	74.82 ± 1.84	69.72 ± 2.07	0.079
E/e	8.87 ± 1.81	19.06 ± 1.07	0*
e (m/s)	0.1 ± 0.001	0.05 ± 0.002	0*
LA (mm)	31.40 ± 2.77	38.37 ± 5.57	0*
LAVI (ml/m^2^)	26.9 ± 3.96	39.6 ± 1.69	0*

*P < 0.05.

There was no significant difference in age, HR, blood pressure, and ESV between the two groups (p > 0.05). Although E/e, e, LA, and LAVI were statistically different in the control and HCM groups, these values had no correlation with METS (p > 0.05). Global work efficiency (GWE) (r = 0.278), GWI (r = 0.391), and GCW (r = 0.372) had positive correlation with METS (all *P*-values < 0.05).

3 Comparison of strain and myocardial work

**FIGURE 1:**
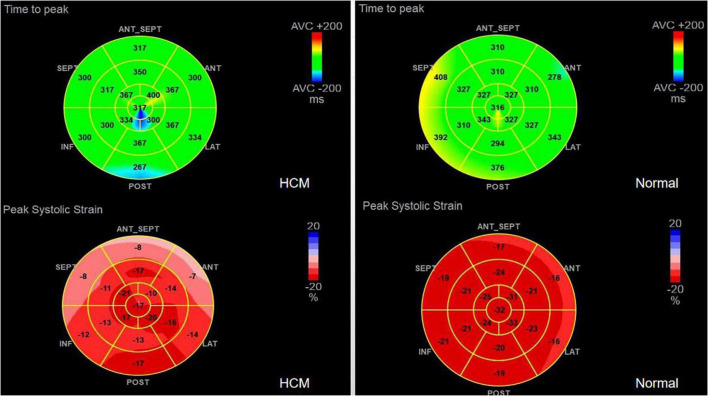
PSD AND PSS in HCM and Normal group.

**FIGURE 2:**
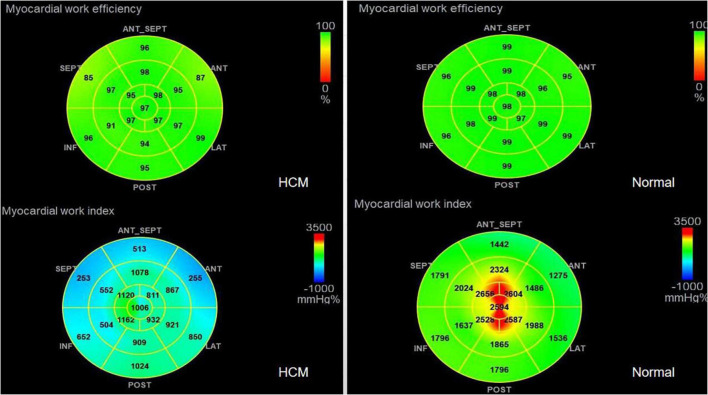
Comparison of GWE and GWI in HCM and Normal group.

**FIGURE 3:**
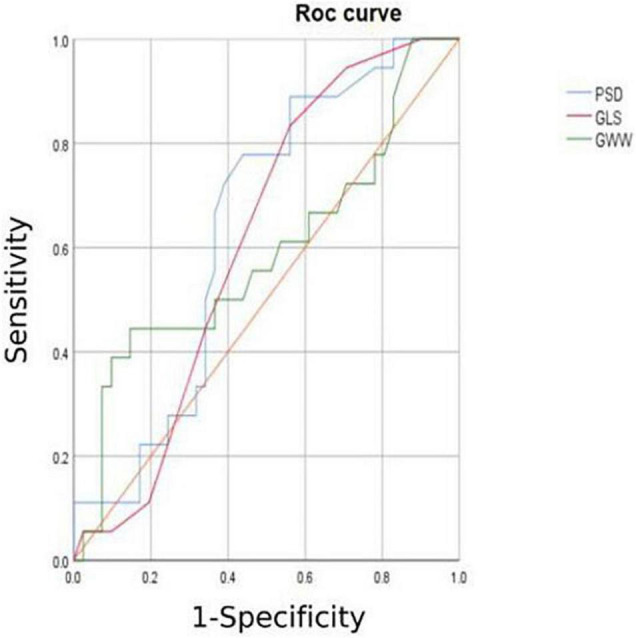
ROC curve of GLS AND PSD.

**FIGURE 4:**
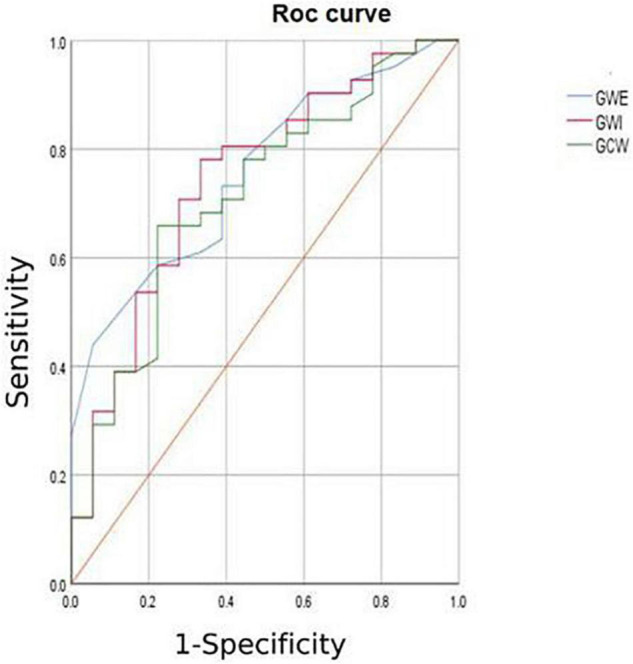
ROC curve of myocardial work.

1. TTPLS: Compared with the normal group, the BLST (396.32 ± 99.9) ms, MLST (396.56 ± 13.87) ms, and ALST (401.74 ± 14.55) ms of the HCM group were significantly prolonged, and the PSD of the HCM group was significantly prolonged and higher than that of the normal group (95.10 ± 8.15 vs. 28.97 ± 1.50) (*p* < 0.01) (Figure 1 and Table 2).
2. LV-GLS in the HCM group was significantly lower than that in the normal group (−17.77 ± 0.20% vs. −21.66 ± 0.42%) (*p* < 0.05) (Figure 1 and Table 2).
3. LV-LPS: An increasing trend was shown in the basal segment (LPS-BL) < intermediate segment (LPS-ML) < apical segment (LPS-AL) for both patients with HCM and controls, although each segment had lower values in the HCM group. The LPS-BL, LPS-ML, and LPS-AL of the HCM group were significantly lower than the corresponding segments of the normal group (−9.34 ± 1.03% vs. −19.46 ± 0.21%), (−11.52 ± 0.83% vs. −20.86 ± 1.98%), and (−14.84 ± 1.37% vs. −25.2 ± 0.73%) (*p* < 0.01) (Table 2).
4. GWE (87.46 ± 6.10%), GWI (1,243.53 ± 424.52 mmHg%), and GCW (1,375.3 ± 436.67 mmHg%) in the HCM group were significantly lower than those in the control group (*p* < 0.01), and only GWW (119.77 ± 13.19 mmHg%) was significantly higher than that in the normal group (*p* < 0.01; Figure 2 and Table 2).
5. In Table 3, a comparison of asymmetric HCM and AHCM found that the METS and LV-GLS of HCM in the asymmetric group were significantly lower than that in the AHCM group, but the PSD was significantly greater than that in the AHCM group. Values of LPS-BL (−13.13 ± 2.51% vs. −10.17 ± 2.20%) in the AHCM group were better than that in the asymmetric HCM group (*p* < 0.05). There were no significant differences between the asymmetric HCM and AHCM groups concerning values for LPS-ML, LPS-AL, GWE, GWI, GCW, and GWW.
6. The HCM group was divided into group A (METS ≤ 6.8) and group B (METS > 6.8) according to METS (8): the results showed that the resting state GWE is less than 92.5%, GWI is less than 1,200 mmHg%, and GCW is less than 1,399 mmHg%, which were helpful for the diagnosis of impaired exercise tolerance (Table 4 and Figures 3, 4).

**TABLE 2:** Comparison of myocardial work and strain between normal group and HCM group.

Variable	Normal group	HCM group	*P*
BLST (ms)	359.86 ± 6.74	396.32 ± 9.99	0.007*
MLST (ms)	344.28 ± 6.73	396.56 ± 13.87	0.002*
ALST (ms)	339.59 ± 6.90	401.74 ± 14.55	0.001*
PSD (ms)	28.97 ± 1.50	95.10 ± 8.15	0*
LV-GLS (%)	−21.66 ± 0.42	−17.77 ± 0.20	0.026*
LPS-BL (%)	−19.46 ± 0.21	−9.34 ± 1.03	0*
LPS-ML (%)	−20.86 ± 1.98	−11.52 ± 0.83	0*
LPS-AL (%)	−25.2 ± 0.73	−14.84 ± 1.37	0*
GWE (%)	97.7 ± 0.16	87.46 ± 6.10	0*
GWI (mmHg%)	2,014.5 ± 355.28	1,243.53 ± 424.52	0*
GCW (mmHg%)	2,309.03 ± 425.77	1,375.3 ± 436.67	0*
GWW (mmHg%)	39.93 ± 3.79	119.77 ± 13.19	0*

*P < 0.05.

**TABLE 3:** Comparison of myocardial work and strain between asymmetric and apical groups.

Variable	Asymmetric HCM (*N* = 34)	Apical HCM (*N* = 31)	*P*
METS	8.91 ± 0.43	10.17 ± 0.12	0.007*
SBP (mmHg)	128.37 ± 2.59	123.60 ± 3.12	0.204
DBP (mmHg)	76.07 ± 1.52	76.03 ± 1.94	0.988
GWE (%)	89.06 ± 1.05	88.13 ± 1.57	0.58
GWI (mmHg%)	1,395.3 ± 88.34	1,264.8 ± 84.43	0.227
GCW (mmHg%)	1,528.13 ± 93.72	1,427.97 ± 84.97	0.37
GWW (mmHg%)	119.53 ± 10.06	128.63 ± 16.95	0.642
LV-GLS (%)	−17.55 ± 0.27	−18.43 ± 0.13	0.007*
LPS-BL (%)	−10.17 ± 2.20	−13.13 ± 2.51	0.022*
LPS-ML (%)	−12.48 ± 3.21	−14.02 ± 3.90	0.320
LPS-AL (%)	−18.89 ± 2.76	−15.17 ± 3.65	0.355
PSD (ms)	115.93 ± 7.92	91.9 ± 8.34	0.033*
BLST (ms)	403.20 ± 27.61	397.54 ± 37.20	0.861
MLST (ms)	405 ± 22.46	390.66 ± 47.75	0.668
ALST (ms)	381.56 ± 44.85	384.81 ± 24.35	0.885

*P < 0.05.

**TABLE 4:** Specificity and sensitivity analysis in HCM.

Variable	AUC	Cut-off value	Sensitivity	Specificity	Asymptotic significance b
GWE (%)	0.645	92.50%	0.898	0.375	0.043*
GWI (mmHg%)	0.784	1,200	0.6738	0.812	0.001*
GCW (mmHg%)	0.751	1,399	0.653	0.812	0.003*
LV-GLS	0.517	/	/	/	0.843

*P < 0.05.

## Discussion

Hypertrophic cardiomyopathy is an autosomal dominant disorder with a high degree of heterogeneity in its clinical presentation. Exertional dyspnea, chest pain, fatigue, and presyncope or syncope are all common in patients with HCM. Possible mechanisms include increased myocardial oxygen consumption and demand after exercise, decreased myocardial blood supply, abnormal vasomotor responses, and vascular remodeling (13). Regardless of symptom status, the ACCF/AHA guidelines recommend lifestyle optimization at the time of HCM diagnosis. Low-intensity aerobic exercise is recommended to achieve and maintain cardiovascular fitness (14). Therefore, the assessment of exercise tolerance is particularly important for patients with HCM. The main findings of this study may be summarized as follows: (1) The left ventricular longitudinal contractility, systolic synchrony, and myocardial function were impaired in patients with HCM in whom the ejection fraction had been preserved in the resting state. (2) Patients with HCM showed decreased MW and work efficiency and increased GWW. (3) Myocardial contractility, systolic synchronization, and exercise tolerance were significantly lower in the patients with asymmetric HCM than in the AHCM group. (4) Values of GWW, GLS, GWI, PSD, GWE, and GCW have utility for the estimation of the intensity range of exercise tolerance.

The sinus node normally sends a discharge impulse down the ventricle through the bundle of His from the endocardium to the epicardium which travels from the apex to the bottom of the heart. Synchronous contraction of the myocardium completes the “pump function.” The quantification of time synchronization of electromechanical conduction (*via* PSD measurement) on the basis of strain may be used to study myocardial contractile function. Haland et al. (15) and Candan et al. (16) showed that the reduction of LV-GLS and the prolongation of PSD in HCM may be helpful for the early identification of the condition requiring implantable cardioverter defibrillator (ICD) treatment. Such measurements are useful to guide clinicians and inform targeted early-stage treatment plans, thus improving patient outcomes. The cardiomyocytes in patients with HCM show a disordered hypertrophic arrangement with the appearance of fibrosis and increased cellular oxygen consumption and metabolic coronary intima damage results (17, 18). Cardiomyocyte hypertrophy is accompanied by fibrosis, effective cardiomyocytes per unit area decrease, and myocardial oxygen consumption increases significantly after exercise; the increase of intramyocardial vascular pressure per unit area and a relative decrease of myocardial blood supply per unit area reduce blood supply and result in decreased myocardial strain and increased systolic heterogeneity among different myocardial segments. This study found that patients with HCM, in whom ejection fraction at rest was normal, had reduced segmental strain and LV-GLS in combination with prolonged TTPLS and PSD. These findings show that left ventricular contraction mechanics and synchronicity have been significantly damaged, suggesting that LVGLS and PSD measurements would detect abnormal left ventricular systolic function in subclinical segments. Such findings are consistent with the results of Panoulas et al. (19). The occurrence of abnormal left ventricular mechanics in HCM with preserved ejection fraction accounts for exercise intolerance. Anatomical studies show that LV-GLS largely depends on the subendocardial layer of the myocardium which is the most sensitive to ischemia (20). A retrospective study involving more than 3,000 patients with HCM showed that abnormal LV-GLS is associated with adverse cardiovascular events and ventricular arrhythmias, accounting, at least in part, for high rates of sudden death in young patients with HCM (21). Increased PSD and the deterioration of synchrony may be related to myocardial scarring and HR. Abd-Elmoniem et al. (22) found that myocardial scarring caused a loss of synchrony by reducing wave propagation speed in the infarct and surrounding areas. Furthermore, exercise increases the HR, shortens the diastolic period, and causes increased myocardial oxygen consumption leading to an imbalance between supply and demand. LPS is obtained by using two-dimensional speckle-tracking imaging (2D-STI) technology, the principle of which relies on spots in the region of interest for use as tissue markers. The software automatically matches the best mode to track and identify changes in the spatial position of the spot signals in the region of interest, calculating the strain of each myocardial segment to represent deformability (23). This study found an increasing trend in the basal segment (BL) < intermediate segment (ML) < apical segment (AL) for both patients with HCM and controls. Each segment produced lower values in the HCM group. Left ventricular contraction proceeds from the apex to the bottom of the heart and BL in the AHCM group > BL in the asymmetric HCM group (*p* < 0.05). During the development of asymmetric HCM, the septum or other ventricular walls are thickened and the ventricular septum not only undertakes the function of left ventricular contraction but also connects the outflow tract and the mitral valve. This process may be influenced by the abnormal hemodynamic impact caused by the hypertrophic myocardium. Wall fibrosis and exercise involvement are more obvious in the case of asymmetric HCM when, especially after exercise, the HR increases and the left ventricle presents a hyperdynamic state. There was no statistical difference between ML and AL for the two groups. Although hypertrophy affects the local myocardial segment, electrical and mechanical effects on cardiomyocytes extend to adjacent regions with delayed electrical activity and reduced mechanical activity.

In combination with decreased coronary perfusion, induced myocardial ischemia and blood flow redistribution, and decreased myocardial strain (24), where all aggravate the impairment of synchrony, the co-existence of these types of damage leads to mutual aggravation. Abnormalities of electromechanical motion reduce the mechanical efficiency of left ventricular ejection, increasing energy loss and ineffective work and reducing overall work and efficiency (25–27). When this situation is prolonged, myocardial contractile function gradually deteriorates and may even lead to heart failure. Schrub et al. (28) and Duchenne et al. (29) found that GWW increased but GWE decreased when the left ventricular PSD was enlarged in HCM and this may be related to the decreased metabolic activity of the ventricular septum. This study also found PSD in asymmetric HCM > PSD in AHCM (115.93 ± 7.92 vs. 91.9 ± 8.34; *p* = 0.033). Moreover, Aalen et al. (30) found that HCM patients with reduced synchrony had a more sensitive ventricular septum and lower exercise tolerance. This finding was confirmed by METS in this study (asymmetric HCM: 8.91 ± 0.43 vs. AHCM: 10.17 ± 0.12; *p* = 0.007). Ma et al. (31) also showed that the incidence of myocardial fibrosis and the proportion of fibrotic myocardial mass were higher in patients with asymmetric HCM than that in those with AHCM which is similar to the current findings. MRI data required for the verification of the degree and extent of myocardial fibrosis were not available for this study. However, this can be inferred from PSD, LV-GLS, and LPS values and the PSD for the asymmetric group was found to be significantly larger than that of the AHCM group. Myocardial fibrosis causes a loss of synchrony by reducing wave propagation speed in fibrotic areas (22), and previous studies have established the molecular basis to be enhanced actin-myosin interaction that delays and weakens cardiomyocyte contraction (32). Thus, as the intensity decreases, the measured LV-GLS decreases. Only eight subjects with HCM enrolled in this study had received cardiac MRI at the time of writing, and statistical analysis could not be completed for the whole sample. It is the intention of the authors to wait until all patients have completed cardiac MRI before adding MRI parameters to the comparison analysis. A statistical analysis of the diagnostic capacity of MW parameters for exercise tolerance was conducted separately in asymmetrical HCM and AHCM. We found that only LV-GLS had statistical differences [area under the curve (AUC) 0.739] in the asymmetric HCM group and only GWI (AUC 0.852) in the AHCM group. Considering the small sample size of METS (less than 6.8) in the respective groups, statistical deviation is possible, precluding a discussion of the diagnostic utility of exercise tolerance by an echocardiographic parameter in the separate cases of asymmetrical and AHCM. However, the enrollment of patients with HCM continues in an attempt to expand the sample size. We hope that follow-up studies can be performed and contributed to the discussion of this component. In general, patients with HCM have worse synchrony, increased GWW, and significantly decreased GCW, GWI, and GWE, and those with asymmetric HCM had lower strain, worse synchrony, and lower METS. Myocardial electrical conduction and mechanical contraction start from the apex and travel to the bottom of the heart. Segmental myocardial strain in the healthy subject shows the trend: BL < ML < AL with the BL being dominated by longitudinal contraction and the apical segment by torsion. The heart of the patient with HCM shows a similar trend but BL strain in the asymmetric HCM group was significantly lower than that in the AHCM group (*p* < 0.05). The balance between asymmetric HCM mechanics and intracardiac hydrodynamics may be lost and hypertrophy of the BL may lead to more turbulent inflow and outflow, resulting in greater energy loss. However, further and more detailed histopathological studies are required for verification. Schrub showed that the GWE of the interventricular septum was the best predictor of exercise capacity and that dispersion was related to the uneven distribution of MW in patients with dilated cardiomyopathy (DCM) (28). Marwick et al. demonstrated that in heart failure with preserved ejection fraction, GCW better-reflected exercise tolerance than GLS: when GCW increased significantly, exercise tolerance improved (33). Selecting for METS greater than or less than 6.8, this study found that AUC values for GWE (0.645), GWI (0.784), and GCW (0.751) were all higher than that for LV-GLS (0.517), in agreement with the above results. When values of GWE were less than 92.5%, GWI less than 1,200 mmHg, and GCW less than 1,399 mmHg, these are reference values with utility in the diagnosis of impaired exercise tolerance and poor prognosis.

## Limitations

This study was limited to a single center. In order to exclude differences caused by ethnicity of the geographical population, the study collected matched healthy control participants from the same area and hospital as enrolled patients with HCM. In addition, the sample size was small and the conclusions require further studies with larger sample sizes or multicenters to make up for current deficiencies. Diastolic function can be reflected by parameters such as E/e, e, and LAVI. Among the 65 patients with HCM, 31 cases had mild mitral regurgitation, 11 had moderate mitral regurgitation, 2 had severe mitral regurgitation, 28 had mild tricuspid regurgitation, and 10 had moderate tricuspid regurgitation. All subjects had non-obstructive HCM but 20 patients also had increased outflow tract velocity. Therefore, the degree of mitral regurgitation, the increased outflow tract velocity, the smaller cardiac chamber caused by ventricular wall hypertrophy, the volume load of the left ventricle, and the high hemodynamic state in the cardiac chamber may all affect E, e, E/e, LA, LAVI, and other parameters for the evaluation of diastolic function. The above parameters were found to have no significant correlation with METS at rest but this finding cannot rule out the existence of a correlation between E, e, E/e, LA, LAVI, the diastolic reserve function of HCM during the exercising state, and METS. Therefore, relevant guidelines (10) suggest that the diastolic evaluation of patients with HCM should be combined with other influencing factors. This study focused on MW, and further diastolic function parameters which were analyzed in our follow-up study have not been included in this report.

## Conclusion

GWI, GWE, and GCW can be safely measured by resting echocardiography to evaluate exercise tolerance in patients with HCM who cannot perform an exercise-based examination. Such measurements provide a basis for clinical decisions regarding exercise and drug prescription.

## Data Availability Statement

The raw data supporting the conclusions of this article will be made available by the authors, without undue reservation.

## Ethics Statement

The studies involving human participants were reviewed and approved by the Human Body Research Institution Committee of Sichuan Provincial People’s Hospital. The patients/participants provided their written informed consent to participate in this study.

## Author Contributions

YS, LY, and CL: conception and design. LY: administrative support. CL: provision of study materials or patients. YS and QP: collection and assembly of data, and data analysis and interpretation. All authors contributed to manuscript writing and final approval of the manuscript.

## Conflict of Interest

The authors declare that the research was conducted in the absence of any commercial or financial relationships that could be construed as a potential conflictof interest.

## Publisher’s Note

All claims expressed in this article are solely those of the authors and do not necessarily represent those of their affiliated organizations, or those of the publisher, the editors and the reviewers. Any product that may be evaluated in this article, or claim that may be made by its manufacturer, is not guaranteed or endorsed by the publisher.
